# Effect of malaria in pregnancy on foetal cortical brain development: a longitudinal observational study

**DOI:** 10.1186/1475-2875-11-222

**Published:** 2012-07-02

**Authors:** Marcus J Rijken, Merel Charlotte de Wit, Eduard JH Mulder, Suporn Kiricharoen, Noaeni Karunkonkowit, Tamalar Paw, Gerard HA Visser, Rose McGready, François H Nosten, Lourens R Pistorius

**Affiliations:** 1 Shoklo Malaria Research Unit, PO Box 46, Mae Sot, Tak 63110, Thailand; 2 Department of Obstetrics, University Medical Center Utrecht, Utrecht, Netherlands; 3 Centre for Tropical Medicine, Churchill Hospital, Oxford, UK; 4 Faculty of Tropical Medicine, Mahidol University, Bangkok, Thailand

**Keywords:** Malaria, Pregnancy, Ultrasonography, Prenatal, Brain, Foetus, Cerebral cortex

## Abstract

**Background:**

Malaria in pregnancy has a negative impact on foetal growth, but it is not known whether this also affects the foetal nervous system. The aim of this study was to examine the effects of malaria on foetal cortex development by three-dimensional ultrasound.

**Methods:**

Brain images were acquired using a portable ultrasound machine and a 3D ultrasound transducer. All recordings were analysed, blinded to clinical data, using the 4D view software package. The foetal supra-tentorial brain volume was determined and cortical development was qualitatively followed by scoring the appearance and development of six sulci. Multilevel analysis was used to study brain volume and cortical development in individual foetuses.

**Results:**

Cortical grading was possible in 161 out of 223 (72%) serial foetal brain images in pregnant women living in a malaria endemic area. There was no difference between foetal cortical development or brain volumes at any time in pregnancy between women with immediately treated malaria infections and non-infected pregnancies.

**Conclusion:**

The percentage of images that could be graded was similar to other neuro-sonographic studies. Maternal malaria does not have a gross effect on foetal brain development, at least in this population, which had access to early detection and effective treatment of malaria.

## Background

Both *Plasmodium vivax* and *Plasmodium falciparum* malaria are associated with maternal and foetal morbidity and mortality [1,2]. Malaria in pregnancy causes a decrease in birth weight by intra-uterine growth restriction (IUGR), preterm delivery, or both [3]. Malaria infection in the first half of pregnancy is associated with a reduction in foetal head diameter [4] and maternal *P. falciparum* malaria changes utero-placental haemodynamics [5]. Whether in-utero exposure to malaria has an effect on the growth and development of the foetal central nervous system is not known.

Studying the pathophysiological consequences of malaria in pregnancy on the foetus has been complicated by unreliable diagnosis of (early) pregnancy infections, difficulties in gestational age (GA) estimation and use of various methods to measure newborn anthropometrics [6]. The primary cortical folding process in the newborn is an early marker for functional neuro-development [7]. Growth restricted foetuses show a faster cortical folding process in comparison to normal foetuses, when measured with magnetic resonance imaging [7] or three-dimensional (3D) ultrasound (C. Businelli, unpublished data). Such increased cortical maturation might be related to the functional disturbances (e.g. lower IQ, attention deficit hyperactivity disorder) found in children affected by IUGR [8,9]. The relatively new technique of 3D-ultrasound imaging of the foetal brain has rarely before been available in malaria endemic areas. The aim of this pilot study was to examine the effects of malaria in pregnancy on foetal brain development. The hypothesis was that foetuses affected by maternal malaria experience growth restriction, including smaller foetal brain volume and an accelerated cortical folding process.

## Methods

### Population

The participants in this study were attending the antenatal clinic (ANC) at Shoklo Malaria Research Unit (SMRU), which is located on the Thai-Burmese border where malaria transmission is low and seasonal [10]. Since 1986, the SMRU runs an ANC programme including weekly malaria screening to detect and treat all parasitaemic episodes during pregnancy to prevent maternal deaths [10]. There is no presumptive treatment of malaria or chemoprophylaxis. Every woman with a malaria episode detected by peripheral smear is immediately treated using WHO protocols: chloroquine for *P.vivax* infections in any trimester and artemisinin-based combination therapy for *P.falciparum*, and quinine-clindamycin for first trimester infections [11]. Routine antenatal ultrasound performed by locally trained health workers commenced in 2001 [12]. All women are encouraged to attend the ANC as early as possible in pregnancy and to deliver at SMRU under the care of Advance Life Support in Obstetrics (ALSO) trained midwives and doctors; those requiring caesarean section are transferred to the nearest Thai hospital. In this study, pregnant women with documented *P. falciparum* or *P. vivax* parasitaemia were included. They were compared with uninfected women matched for foetal gender, parity, and maternal age (within five years).

### Ultrasound scans

Ultrasound scans were performed trans-abdominally using a General Electric Voluson i (GE Healthcare, Austria) with a RAB2-5-RS, 2–5 MHz real-time 4D probe. The machine was housed in a dedicated air-conditioned room. All scans were obtained by locally trained sonographers specifically skilled in advanced foetal growth scanning (SK and NK) or a resident in obstetrics certified in antenatal ultrasound scanning (MJR), with regular internal quality control at SMRU. In addition, all images were sent for external quality control to the INTERGROWTH-21st Project team at the University of Oxford [13]. Foetal crown rump length (CRL) between 9^+0^ and 13^+6^ weeks was used to define gestational age (GA). Thereafter, women were invited to attend a foetal growth scan every five weeks until delivery to take 2D foetal biometric measurements and 3D sweeps, including the foetal head at the mid-ventricular plane. The volume box size and sweep angle were adapted to include the entire head. Care was taken to minimize movement artifacts. Once the scan was complete, volume data were stored for later analysis.

### Definitions

Malaria was diagnosed by Giemsa stained thick and thin blood films; 200 fields on the thick film were read before being declared negative. Severe malaria was defined according to WHO treatment guidelines [11]. Birth weight analysis was confined to life born, congenitally normal, singleton infants weighed in the first 24 hrs of life. Prematurity was defined as delivery before 37 ^+ 0^ weeks’ gestation. Birth weight, length, and head circumference were measured twice by the trained and quality controlled anthropometry team on electronic Seca baby scales (Model 376, accuracy 10 grams), a Harpenden Infantometer with digital counter readings to one mm, or Seca Head Circumference Tape accurate to one mm, respectively, the first two being calibrated twice a week [6]. All ultrasound and anthropometric methods were identical to the INTERGROWTH-21^st^ study protocol [13].

One author (MCW), blinded to any clinical data, analysed all recorded volumes using the 4D view software package, version 9.1 (GE healthcare). The foetal supra-tentorial brain volume was determined and cortical development was qualitatively followed by scoring the appearance and development of six sulci (the Sylvian fissure and the superior temporal, central, parieto-occipital, calcarine and cingulate sulcus) [14]. A grading system was used to systematically assess the development of every sulcus independently. The depth and ramification of the specific sulcus was scored in a range from zero to five, where zero equals “not visible” and five “fully developed”, as described previously [14]. Sulci on both the left and right side of the brain were graded, and the mean grade was used for analysis.

### Statistical analysis

Clinical data and the results of the ultrasound scans were entered into a Microsoft Access database and analysed using SPSS version 17 for Windows. The Mann–Whitney, Chi-square or Fisher’s exact test were used for comparison of ranks or categorical data, as appropriate. Multilevel analysis was used to study brain volume and cortical development in individual foetuses, with grades considered as a continuous variable [14]. The significance level was set at α = 0.05.

### Ethical approval

This study was part of a larger foetal growth project (ClinicalTrials.gov Identifier: NCT00840502), approved and yearly renewed by the Ethics Committees of Oxford (OxTREC (14–08)) and Mahidol (TMEC 2008–028) universities. All women provided written informed consent.

## Results

**Figure 1:**
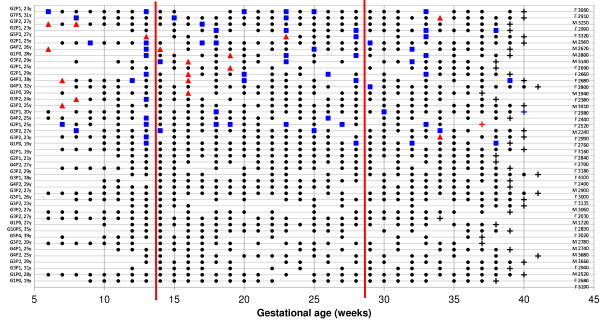
**Summary of morbidity.** The x-axis is the gestational age in weeks. Each pregnant woman is shown as a horizontal line; the upper 22 horizontal lines represent women with malaria infections. The left column indicates the parity and the age of each pregnant woman, the right column shows the sex and weight of the newborn. The vertical red lines mark the first, second and third trimester. Blue boxes are *P. vivax* infections, and red triangles *P. falciparum* infections. Each black dot is an antenatal clinic consultation including a malaria screening, a cross (+) is a delivery, and could have a blue or red colour indicating *P. vivax* or *P. falciparum* infection at delivery, respectively. Abbreviations: F = Female, G = Gravida, M = Male, P = Parity, Y = Year.

In total 215 women were recruited between February 2009 and August 2010. Of these 22 women were diagnosed with malaria infections: 14 were infected with *P. falciparum* malaria and eight women with *P. vivax*. The characteristics of both infected and non-infected women are shown in Table 1. The frequency and timing of the infections are shown in Figure 1. Most malaria episodes were uncomplicated (77/78; 98.7%). One pregnant woman had severe malaria during labour. There were more anaemic women in the malaria group, as expected. Three of these women required a blood transfusion. Other morbidities in pregnancy are summarized in Table 1.

**Table 1:** Maternal and newborn characteristics

	**No malaria *(n = 22) ***	**Malaria *(n = 22) ***	**P**
**Pregnant women**			
Age (yrs)	27.0 (19 – 39)	25.5 (19 – 38)	0.23
Nulliparous	3 (14)	3 (14)	1.0
Gravida	3 (1 – 10)	3 (1 – 7)	0.36
Parity	2 (0 – 5)	2 (0 – 5)	0.82
Height (m)	1.53 (1.46 – 1.64)	1.53 (1.44 – 1.61)	0.71
Weight (kg)	50.5 (39 – 70)	46.5 (39 – 61)	0.20
Weight gain (kg)	7.5 (−2 – 17)	9 (4 – 15)	0.76
BMI (kg m^-2^)	21.5 (17.8 – 30.2)	20.1 (17.1 – 27.6)	0.15
MUAC (cm)	25.6 (14.5 – 32.0)	25.0 (20.6 – 30.6)	0.81
Smoking	3 (14)	7 (32)	0.28
NOC	25.5 (12–34)	26.5 (15–33)	0.48
Father’s age	29.5 (19–43)	29.5 (24 – 38)	0.80
Other infections	3 (14)	5 (23)	0.70
Anaemia	7 (32)	14 (64)	0.07
Severe anaemia	0 (0)	3 (14)	0.23
Early pre-eclampsia	2 (9)	2 (9)	1.0
**Newborns**			
Gestational Age (days)	277 (241 – 295)	279 (261 – 293)	0.58
Sex (% boys)	36.4%	36.4%	1.0
Weight (grams)*	2840 (1720 – 3660)	2685 (1940 – 3410)	0.18
Length (cm)*	49.0 (44.6 – 51.0)	49.4 (45.0 – 51.3)	0.88
Head circumference (cm)*	32.0 (29.5 – 39.3)	32.0 (31.0 – 35.1)	0.53

Data are presented as median (range) or as number (%). MUAC mid upper arm circumference, NOC number of consultations. * Data shown of newborns weighed within 24 hours of delivery; 17 and 18 in the no malaria and malaria group, respectively.

### Cortical maturation and supra-tentorial brain volume

**Figure 2:**
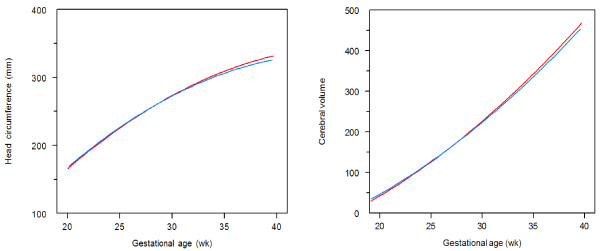
**Head circumference and cerebral volume.** The x-axis is the gestational age in weeks; the y-axis represents the head circumference in millimetres or cerebral volume in centilitres. The blue lines represent foetuses from women with malaria infections (n = 22) and the red lines show foetuses from women without malaria infections (n = 22) in pregnancy.

**Figure 3:**
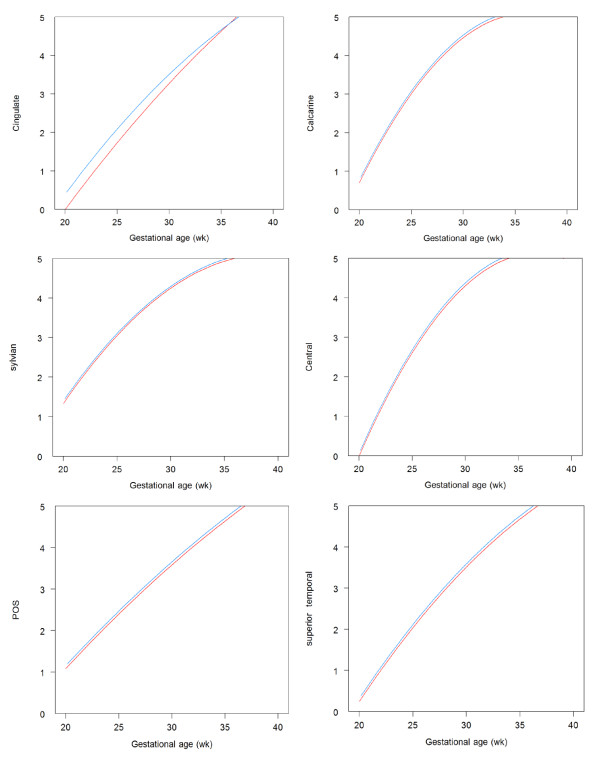
**Development of foetal cortex (Sylvian fissure, superior temporal, central, parieto-occipital, calcarine and cingulate sulcus).** The x-axis is the gestational age in weeks; the y-axis represents the grading of the Sylvian fissure, superior temporal, central, parieto-occipital, calcarine and cingulate sulcus. The blue lines represent foetuses from women with malaria infections (n = 22) and the red lines show foetuses from women without malaria infections (n = 22) in pregnancy.

In total, 223 brain images were analysed: 113 in the malaria group and 110 in the non-infected group. The median number of brain images per foetus was five (range 4–6) and did not differ between both groups. In the malaria affected group, 73.4% (83/113) of images could be visualized well enough to grade them reliably. In the non-infected group this percentage was similar (70.9%; 78/110), p = 0.78. The presence of reverberation artefacts was the most important reason for the inability to visualize a sulcus in the hemisphere closest to the abdominal wall. Furthermore, in early pregnancies, foetal motion artefacts often troubled visualization. The head circumference and brain volumes between malaria infected and uninfected pregnancies, which were not significantly different, are illustrated in Figure 2. Table 2 shows the mean GA at first appearance and full foetal development per sulcus. All six sulci developed similarly between the two groups (Figure 3, Table 2). The median number of days between the first appearance of any sulcus and the first fully matured sulcus in the same foetus was 105 for both malaria [range 79–133] and non-malaria [range 70–139] groups (p = 0.21). Only the cingulate sulcus initially matured significantly faster in foetuses affected by maternal malaria in pregnancy in comparison to foetuses of malaria-free pregnancies (Figure 3). There was no significant association between the number of episodes, species, timing of infection, or number of previous pregnancies and cortical maturation or volumes.

**Table 2:** Time (in days) of first appearance, interval and full development of cortical sulci

**Sulcus**	**Group**	**First appearance**	**p**	**Maximal grade**	**p**
Sylvian	Malaria	115 (99 – 131)	0.539	230 (204 – 268)	0.772
	Control	117 (101 – 134)		231 (204 – 272)	
Superior Temporal	Malaria	145 (118 – 179)	0.362	243 (211–268)	0.455
	Control	150 (112 – 177)		238 (172 – 263)	
Parieto-occipital	Malaria	123 (99 – 147)	0.422	240 (204–277)	0.455
	Control	120 (98 – 146)		244 (216 – 274)	
Central	Malaria	145 (104 – 167)	0.429	221 (201 – 268)	0.586
	Control	150 (112 – 169)		219 (191 – 241)	
Calcarine	Malaria	126 (99 – 151)	0.282	226 (204 – 226)	0.706
	Control	133 (109 – 172)		224 (202 – 263)	
Cingulate	Malaria	145 (106 – 179)	0.156	248 (217 – 277)	0.600
	Control	154 (134 – 179)		244 (211–274)	

Data are presented as median (range).

### Newborns

Eleven (25%) women delivered at home, one (2%) underwent a caesarean section because of prolonged labour and the remaining 32 (73%) delivered in the SMRU clinic. There were no stillbirths, and all newborns appeared congenitally normal, although one infant from the non- malaria group was diagnosed with congenital heart disease later in life. Overall, the median birth weight was 2,780 [range 1,720 – 3,660] grams in newborns weighted within 24 hours after delivery (n = 35), and the gestational age at delivery was 39^+3^ [range 33^+3^ – 41^+2^] weeks, including one premature neonate. All anthropometric measurements in the newborn were similar between the two groups (Table 1).

## Discussion

In this study, the foetal brain volumes and foetal cortex development were compared between women with and without malaria. Although the images of this study were not primarily obtained for neuro-sonographic evaluations and 3D ultrasound exposure time was limited, seventy percent of sulci could be visualized well enough to be graded. This percentage is similar to a previous study in healthy volunteers, where ultrasound scans were performed by a neuro-sonography expert [14]. In the busy malaria clinics, there was no possibility for real-time evaluation of the quality of obtained images. This is the first study to address the effect of maternal malaria on foetal cortical development or supra-tentorial volume. No difference in brain volume expansion or foetal cortical folding in general at any time in pregnancy between women with immediately treated malaria infections and non-infected pregnancies was detected. Of all graded sulci, only the cingulate sulcus matured faster in foetuses of women affected by malaria during pregnancy. The significance of a difference in maturation of a single sulcus has to be interpreted cautiously in the analysis of the general cortical development. Although the sample size of this pilot study is small, the blinding of the sonographers and observers to the malaria status of the mother, and the well matched groups may allow preliminary conclusions that maternal malaria does not have a gross effect on foetal brain development, at least in this population which had access to early detection and effective treatment of malaria.

The timing of malaria infection has an impact on the growth and development of the foetus [15]. The small sample size did not allow sub-analysis of groups infected in certain trimesters in pregnancy, nor of effect of symptoms or malaria species separately, but most women (73%, 16/22) were infected as early as the first trimester and most women had malaria infections throughout pregnancy. Similar studies with larger sample sizes in different malaria endemic settings may be needed to confirm these findings. Although three dimensional ultrasound machines are delicate and expensive and not available in most malaria endemic settings, this tool may be helpful in determining the impact of malaria on the foetal nervous system and indicating the newborns neurodevelopment.

In conclusion, maternal malaria does not have a gross effect on foetal brain development, at least in this population, which had access to early detection and effective treatment of malaria.

## Competing interest

The authors declare that they have no competing interests.

## Authors’ contributions

MJR designed the study, carried out the ultrasound scanning, performed the statistical analysis and drafted the manuscript. MCW performed the brain volume measurements and cortex grading, performed the statistical analysis and drafted the manuscript. EJHM carried out the statistical analysis and helped to draft the manuscript. SK, NK and TP performed the ultrasound scanning, organized and coordinated the study on site. GHV participated in the design of the study and revision of the manuscript. RMG participated in the design and coordination of the study, helped in the statistical analysis and revised the manuscript. FN conceived of the study and participated in the design and coordination of the study, and revised the manuscript. LRP participated in the design and analysis of the study, organized the image analysis and helped to draft and revised the manuscript. All authors read and approved the final manuscript.
